# Nutzung und Akzeptanz webbasierter Angebote zur Alkoholabstinenz

**DOI:** 10.1007/s00115-022-01385-0

**Published:** 2022-09-13

**Authors:** Nathalie Stüben, Andreas G. Franke, Michael Soyka

**Affiliations:** 1 Klinik und Poliklinik für Psychiatrie und Psychotherapie, Ludwig-Maximilians-Universität München, Nußbaumstr. 7, 80336 München, Deutschland; 2 Hochschule der Bundesagentur für Arbeit, Seckenheimer Landstr. 16, 68163 Mannheim, Deutschland; 3 Nathalie Stüben GmbH, Spinnereiinsel 3a, 83059 Kolbermoor, Deutschland

**Keywords:** Alkohol, Alkoholabhängigkeit, eHealth, Internet, Frühintervention, Therapie, Alcohol, Alcohol dependence, eHealth, Internet, Early intervention, Therapy

## Abstract

**Einleitung:**

Die Prävalenzraten für Alkoholgebrauchsstörungen liegen in Deutschland bei ca. 6 %, alkoholabhängig sind ca. 3 %. Nur ca. 10 % der Patienten befinden sich in suchtmedizinischer Therapie. In der Früherkennung und -intervention besteht zudem eine deutliche Unterversorgung. Eine bislang nicht evaluierte Ergänzung zu bestehenden Interventionsangeboten ist der außerhalb der professionellen Suchthilfe von einer ehemaligen Betroffenen entwickelte Internetauftritt „Ohne Alkohol mit Nathalie“ (OAmN). Die vorliegende Pilotstudie hatte zum Ziel, herauszufinden, ob die Nutzer der OAmN-Angebote zu jenen zählen, die bisher vom Suchthilfesystem nicht erreicht werden konnten.

**Methoden:**

Innerhalb von vier Wochen wurden Nutzer auf vier verschiedenen OAmN-Kanälen dazu aufgerufen, sich an einer anonymen Befragung zu beteiligen. Ein Link führte jeweils zu einem webbasierten Fragebogen. Dieser umfasste offene, geschlossene sowie Multiple-Choice-Fragen zum Konsummuster von Alkohol und bisherigen Therapiebemühungen.

**Ergebnisse:**

Von 2022 Teilnehmern gaben 84,3 % (*n* = 1705) an, ein „Alkoholproblem“ zu haben oder es gehabt zu haben. Davon hatten 17,7 % (*n* = 302) die Diagnose einer Alkoholabhängigkeit und 21 % (*n* = 529) Therapieerfahrung. Sistiert hatten ihren Alkoholkonsum zum Zeitpunkt der Befragung 85,5 % (*n* = 1457) der Betroffenen. Die meisten davon (48,5 %, *n* = 705) mithilfe von OAmN. 97,5 % (*n* = 1662) waren während ihrer Konsumzeit berufstätig und haben aus eigener Sicht zu 34,3 % (*n* = 570) „sehr gut“, zu 43,2 % (*n* = 718) „gut“ funktioniert.

**Diskussion:**

Die Pilotstudie zeigt, dass webbasierte Angebote wie OAmN Menschen erreichen können, die durch Angebote des etablierten Suchthilfesystems nicht erreicht werden, obwohl eine alkoholbezogene Störung vorliegt und eine Bereitschaft zur Verhaltensänderung im Hinblick auf den Alkoholkonsum besteht.

**Zusatzmaterial online:**

Die Onlineversion dieses Beitrags (10.1007/s00115-022-01385-0) enthält einen zusätzlichen Fragebogen. Beitrag und Zusatzmaterial stehen Ihnen auf www.springermedizin.de zur Verfügung. Bitte geben Sie dort den Beitragstitel in die Suche ein, das Zusatzmaterial finden Sie beim Beitrag unter „Ergänzende Inhalte“.

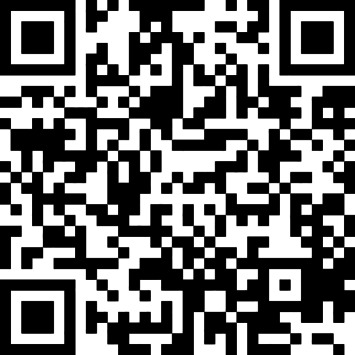

## Abstract (en)

**Introduction:**

In Germany, the prevalence rates for alcohol use disorders amount to approx. 6%, while about 3% are diagnosed as being alcohol dependent. Only 10% of the patients are undergoing treatment. There are apparent deficits with respect to early interventions. The internet presence of “Ohne Alkohol mit Nathalie” (OAmN) (Abstinence with Nathalie) ameliorates options for early treatment interventions using a web-based design; however, this intervention has not been evaluated to date, especially with respect to previous treatments.

**Methods:**

Over a 4-week period, 4 different channels of OAmN posted announcements for a survey participation introducing a link leading to a web-based survey questionnaire on the domain oamn.jetzt. The questionnaire offered open and closed as well as multiple choice questions regarding alcohol use patterns and attempts to change the problematic drinking behavior.

**Results:**

Out of 2022 participants 84.3% (*n* = 1705) stated to have or have had a problem with alcohol use, 17.7% (*n* = 302) had a diagnosis of alcohol dependence by a physician or psychologist and only 21% (*n* = 529) had been in therapy before. The majority of responders (85.5%, *n* = 1457) had stopped alcohol use before participating in the survey. Most of them (48.5%, *n* = 705) were assisted by OAmN, 97.5% (*n* = 1662) had been employed while having the abovementioned problem use of alcohol, 34.3% (*n* = 570) rated their job performance as “very good” and 43.2% (*n* = 718) as “good”.

**Discussion:**

This pilot study revealed that OAmN can reach people affected by problematic drinking behavior who had not been in contact with the professional medical system for addiction treatment despite having a problematic alcohol use combined with the willingness to quit.

Bei Menschen mit Alkoholgebrauchsstörungen besteht in Deutschland eine deutliche therapeutische Unterversorgung. Von jenen, die bereits abhängig sind, befinden sich nur rund 10 % in Therapie. Ein noch größerer Handlungsbedarf besteht im Bereich Früherkennung und -intervention. Webbasierte Angebote könnten diese Lücke schließen.

## Einleitung

In Deutschland liegen die Prävalenzraten für Alkoholgebrauchsstörungen bei ca. 6 %; als alkoholabhängig gelten 3,1 % der 18- bis 64-Jährigen; weitere 2,8 % betreiben Alkoholmissbrauch [5]. Neben volkswirtschaftlichen Schäden führt dieses weit verbreitete Konsummuster auf individueller Ebene zu psychischen und physischen Krankheiten, die durch Abstinenz verhindert werden könnten [3, 7, 22].

Ohne Therapie ist die Prognose von Alkoholgebrauchsstörungen meist ungünstig [6, 17]. In den USA sterben beispielsweise jährlich annähernd 88.000 Menschen auf Grund ihres Alkoholkonsums [16], in Deutschland wird die Zahl der alkoholassoziierten Todesfälle mit 74.000 angegeben [14]. Dennoch erreichen die aktuellen Therapieangebote hierzulande nur 10 % der Betroffenen [21]. Die meisten kommen erst nach vielen Jahren der Erkrankung in suchtmedizinische Behandlung [27]. Noch schlechter sieht es bei jenen aus, die eine Alkoholabhängigkeit entwickeln: „Insbesondere in den Bereichen der Früherkennung und Frühintervention besteht für Menschen mit alkoholbezogenen Störungen bislang eine deutliche Unterversorgung“ [27]. Die aktuelle S3-Leitlinie besagt: „Die genannte Unterversorgung geht allerdings nur zum Teil auf die oben schon angesprochenen Defizite auf der Angebotsseite zurück. Viele Betroffene sind unsicher und schrecken gerade zu Beginn einer Abhängigkeit vor dem Aufsuchen einer Beratung und Behandlung zurück“ [14]. Eine signifikante Anzahl von Patienten kommt somit nicht ins professionelle Suchthilfesystem.

Es bedarf daher niederschwelligerer und alternativer Interventionsangebote, die Betroffene in ihrem Alltag erreichen und unterstützen, weil es „[b]edeutsam ist, dass Menschen mit alkoholbezogenen Störungen möglichst frühzeitig und nahtlos zielgerichtete Hilfe erhalten“ [14]. Hier können webbasierte „e“-Interventionen eine entscheidende Rolle spielen, wozu eine sehr heterogene Gruppe von Maßnahmen zählt [4, 10], darunter Aufklärung und Information, strukturierte Therapieangebote sowie – im weitesten Sinne – Interventionen von Selbsthilfegruppen. Eine vor Kurzem veröffentlichte skandinavische Studie zeigte, dass Onlineinterventionen gerade aufgrund ihrer Anonymität und Verfügbarkeit gewählt wurden [8]. Dabei wurde der Aspekt der persönlichen Identifizierung mit den Inhalten als besonders wichtig angegeben, um die Teilnehmer zur Reflektion anzuregen und Abstinenz zu erreichen [8]. Eine aktuelle systematische Literaturübersicht, die den PRISMA-Guidelines entsprach, zu verschiedenen Suchterkrankungen (Alkoholmissbrauch, Glücksspiel, „binge eating disorder“) zeigte, dass sich von 32 Studien zu Alkoholmissbrauch zwei Drittel als effektiv erwiesen, wobei wiederum zwei Drittel der erfolgreichen Studien einen hohen Qualitätsstandard durch vorherige Entwicklung eines „quality assessment tool“ („selection bias of patients“, „study design“, „blinding“, „drop out rate“ etc.) aufwiesen [10].

Eine stark genutzte deutschsprachige Maßnahme ist das webbasierte Angebot „Ohne Alkohol mit Nathalie“ (OAmN) von Nathalie Stüben, die selbst betroffen war und ihre Alkoholabhängigkeit eigenständig überwunden hat. Ihr Angebot existiert seit Oktober 2019 und gliedert sich in zwei Bereiche: Öffentlichkeitsarbeit und Onlineprogramme. Ersterer umfasst Aufklärungsarbeit (u. a. via Newsletter, Instagram, Podcast, YouTube und klassischer Pressearbeit). Mit den Onlineprogrammen *Die ersten 30 Tage ohne Alkohol* und *Abstinenz stabilisieren* werden Betroffene im „Graubereich“ zwischen „Genusstrinken“ und (körperlicher) Abhängigkeit dabei unterstützt, ein Leben ohne Alkohol zu führen – und das als Gewinn zu betrachten (im Sinne einer zufriedenen Abstinenz). Die Programme kombinieren Wissensvermittlung und Motivation durch persönliche Videoansprache, Begleitmaterialien und eine moderierte Onlinegruppe, in der die Betroffenen sich austauschen können. Die vorliegende Pilotstudie hatte zum Ziel, herauszufinden, ob die Nutzer der OAmN-Angebote zu jenen zählen, die bisher vom Suchthilfesystem nicht erreicht werden konnten.

## Methoden

Die anonyme Onlinebefragung stand potenziellen Teilnehmern vom 01. bis 31.07.2021 zur Verfügung. Diese wurden über vier OAmN-Kanäle darüber informiert:

1. am 01.07.2021 im In- und Outro des Podcasts „Ohne Alkohol mit Nathalie“, der bis zum 31.07. insgesamt 5865 Mal heruntergeladen wurde; hinzu kommen 2985 Streams auf der Plattform „Spotify“,
2. am 01.07. wurde ein Teilnahmeaufruf in der moderierten Onlinegruppe „gepostet“, die zu diesem Zeitpunkt 1046 Nutzer zählte,
3. am 02.07. lief der Aufruf im #nüchtern-Newsletter, der bis zum 31.07. von insgesamt 5557 Abonnenten geöffnet wurde – 1392 davon klickten in dieser Zeit auf den Link zur Umfrage,
4. am 10.07. wurde der Aufruf in einer „Insta-Story“ mit damals 5200 Followern geteilt.

In allen Fällen führte ein Link zur Website „oamn.jetzt“, wo die Umfrage durchgeführt werden konnte.

Aufgesetzt wurde die Onlineumfrage mit dem Tool „Typeform“. Die Probanden wurden bereits in den Teilnahmeaufrufen über Hintergründe, Zweck und Anonymität aufgeklärt sowie auf die Freiwilligkeit der Teilnahme hingewiesen. Unabhängige Variablen wie Alter und Geschlecht wurden zugunsten der Anonymität der Probanden nicht erhoben. Der Fragebogen (siehe elektronisches Zusatzmaterial) umfasste offene und geschlossene sowie Multiple-Choice-Fragen zum Konsummuster von Alkohol und zu bislang unternommenen Bemühungen, den Alkoholkonsum zu sistieren. Es wurden nur die Daten derjenigen zur Auswertung herangezogen, die der Datenschutzerklärung über die Nutzung der Daten zu rein wissenschaftlichen Zwecken am Ende des Fragebogens zugestimmt hatten. Die Datenauswertung erfolgte mit SPSS Statistics Version 28.0.1.0 (Armonk, NY, USA).

## Ergebnisse

Die Website mit dem Onlinefragebogen wurde im o. g. Zeitraum 3080 Mal besucht, der Survey wurde 2549 Mal gestartet und 2022 Mal abgeschlossen (Responserate: 79,3 %). 84,3 % (*n* = 1705) der Teilnehmer gaben an, selbst von einer Alkoholgebrauchsstörung betroffen gewesen oder (immer) noch betroffen zu sein. 7,5 % (*n* = 152) gaben an, die Angebote von OAmN zu nutzen, weil eine nahestehende Person betroffen sei. 8,2 % (*n* = 165) gaben an, die Angebote aus Interesse am Thema zu nutzen (Tab. 1).

**Table 1:** 

	(*n*)	(%)
Selbst betroffen (hat/te ein Alkoholproblem)	1705	84,3
Nicht selbst betroffen – aber nahestehende Person	152	7,5
Nicht selbst betroffen – aber Interesse am Thema	165	8,2
Gesamt	2022	100,0

**Figure 1:**
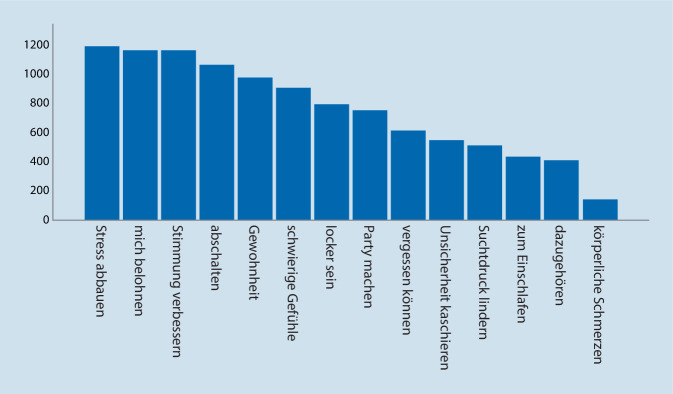

Die Gruppe der Selbstbetroffenen (*n* = 1705) trank aus unterschiedlichen Gründen, von denen Stressabbau (*n* = 1174, 69 %), Stimmungsverbesserung (*n* = 1154, 67,8 %) und Eigenbelohnung (*n* = 1153, 67,8 %) die führenden waren. Weitere Gründe sind Abb. 1 zu entnehmen (Mehrfachantworten waren möglich).

In der Gruppe der Betroffenen (*n* = 1705) ist in 17,7 % (*n* = 302) der Fälle eine Alkoholabhängigkeit von einem Arzt oder Psychologen diagnostiziert worden. Bei der großen Mehrheit von 82,3 % (*n* = 1403) wurde entsprechend keine Abhängigkeit diagnostiziert.

Therapieerfahrung hat die Mehrheit der Selbstbetroffenen nicht (69 %, *n* = 1176). Von den 31 % (*n* = 529) der Therapieerfahrenen haben 58,9 % (*n* = 312) eine ambulante, 17,3 % (*n* = 92) eine stationäre und 23,6 % (*n* = 125) sowohl eine ambulante als auch eine stationäre Therapie absolviert – wobei die offizielle Ursache für die Therapie nicht zwangsläufig Abhängigkeit war, sondern z. B. auch Depressionen oder Angststörungen. Von den Betroffenen ohne Therapieerfahrung (69 %, *n* = 1176) planten nur 7,7 % (*n* = 90) suchttherapeutische Maßnahmen im eigentlichen Sinne.

Gleichzeitig haben von den Betroffenen zum Zeitpunkt der Befragung 85,5 % (*n* = 1457) bereits aufgehört zu trinken; 22 % (*n* = 321) gaben an, „ganz frisch“ den Alkoholkonsum sistiert zu haben, 12,8 % (*n* = 187) lebten seit über einem Monat abstinent, 15,3 % (*n* = 223) seit über drei Monaten, 18,5 % (*n* = 269) seit über sechs Monaten und 31,4 % (*n* = 457) seit über einem Jahr.

Bei der Frage nach ihrem Weg in die Abstinenz gaben die Teilnehmer am häufigsten an, es mithilfe der Angebote von OAmN geschafft zu haben. Weitere Unterstützungsmaßnahmen sind Tab. 2 zu entnehmen.

**Table 2:** 

	(*n*)	%
OAmN	705	48,8
Ärztliche/therapeutische Hilfe	174	12,0
Selbsthilfegruppe	62	4,3
Ohne eine der ersten drei Optionen	501	34,7
Noch nicht geschafft, aber angefangen	111	7,7
Gesamt	1553	107,5

Von den Teilnehmern, die es mithilfe von OAmN geschafft hatten, abstinent zu werden (*n* = 705), beanspruchten 12,6 % (*n* = 89) noch weitere Hilfsangebote: 7,6 % (*n* = 54) nahmen zusätzlich ärztliche und/oder (psycho-/sucht-)therapeutische Hilfe in Anspruch, 3,4 % (*n* = 24) besuchten zusätzlich eine Selbsthilfegruppe. 1,5 % (*n* = 11) haben durch OAmN angefangen mit dem Trinken aufzuhören, würden von sich aber noch nicht sagen, es geschafft zu haben.

Für die folgenden Auswertungen wurden jene berücksichtigt, die selbst betroffen sind (*n* = 1705) und ihren Alkoholkonsum bereits sistiert haben (*n* = 1457).

Bei 35,3 % (*n* = 514) handelt es sich um den ersten Versuch, mit dem Trinken aufzuhören. 32,9 % (*n* = 479) gaben an, dies schon mehrfach versucht zu haben, 31,8 % (*n* = 464) hatten schon „unzählige“ Versuche der Abstinenz unternommen.

Insgesamt 36,7 % (*n* = 534) sind während ihres pathologischen Konsummusters von Alkohol nicht auf ihr Alkoholproblem angesprochen worden, und wenn, geschah dies durch die Familie (32,1 %, *n* = 468), Freunde/Bekannte (23,1 %, *n* = 338) oder Partner/in (39,3 %, *n* = 574), Ärzte/Therapeuten (5,6 %, *n* = 82) oder im beruflichen Umfeld (5,3 %, *n* = 77).

Insgesamt 97,5 % (*n* = 1421) waren trotz Alkoholproblem bzw. während des vorliegenden Alkoholproblems berufstätig; 21,2 % (*n* = 310) in Teilzeit und 76,3 % (*n* = 1111) in Vollzeit. Der Großteil der Gruppe der Berufstätigen (*n* = 1421) hat ihrer subjektiven Einschätzung nach im Beruf „funktioniert“ – 34,3 % (*n* = 487) „sehr gut“, 43,2 % (*n* = 614) „gut“, 17 % (*n* = 242) „befriedigend“ und 3,3 % (*n* = 47) „ausreichend“. Nur 0,4 % (*n* = 6) beurteilten ihre berufliche Funktionsfähigkeit als „mangelhaft“ bzw. 1,8 % (*n* = 25) als „ungenügend“.

Zudem wurde dieselbe Gruppe (*n* = 1457) gefragt, ob sie schon vor der Coronavirus-Pandemie abstinent war: 75,4 % (*n* = 1099) hatten während der Pandemie aufgehört zu trinken, 24,6 % (*n* = 358) davor. Davon gaben 85,8 % (*n* = 307) an, dass ihnen die Abstinenz durch die Pandemie nicht schwerer gefallen war.

Die Probandengruppe, die sich selbst als „nicht selbst betroffen“ einstufte, sondern OAmN-Angebote nutzt, weil eine nahestehende Person ein Alkoholproblem hat (*n* = 152) oder weil sie sich für das Thema interessiert (*n* = 165), wurde bei der Auswertung zusammengefasst (*n* = 317).

Davon gaben 25,9 % (*n* = 82) an, selbst keinen Alkohol zu trinken. Jene 74,1 %, die selbst Alkohol trinken (*n* = 235), befinden sich mit der angegebenen Trinkmenge und -häufigkeit nur zum Teil eindeutig unterhalb der Schwelle zum sog. riskanten Konsum. Die Mehrzahl der Antworten weist bereits auf ein riskantes Trinkverhalten hin, darunter solche wie „regelmäßig abends zwischen 1 und 3 l Bier“, „2- bis 3‑mal Woche, jeweils ungefähr das Doppelte der empfohlenen Höchstmenge/Tag“ oder auch „täglich 2 bis 3 Gläser Wein“.

## Diskussion

Die Ergebnisse dieser Pilotstudie zur Nutzung und Akzeptanz einer webbasierten Intervention bei Menschen mit Alkoholproblem, die außerhalb der professionellen Suchthilfe entwickelt wurde, haben Folgendes gezeigt: Das Interesse an solchen Angeboten ist groß, die Nutzerzahlen hoch. Auch das Interesse, an anonymisierten Befragungen teilzunehmen, ist erheblich. Die meisten Teilnehmer und Rezipienten sind selbst Betroffene, die eigentliche Zielgruppe wird also erreicht. Viele davon haben sich kaum oder noch nie in Behandlung befunden, hier wird also eine neue Zielgruppe erreicht. Zudem lässt sich vermuten, dass die meisten der erfolgreich angesprochenen Teilnehmer sozial eher gut integriert (hohe Anzahl von Erwerbstätigen) und abstinenzmotiviert sind. Jedenfalls ist die Bereitschaft zur Verhaltensänderung im Hinblick auf den Alkoholkonsum hoch.

Lange Zeit dominierten in der Alkoholtherapie Selbsthilfeangebote. Mittlerweile ist die Therapieforschung und Literatur zu Behandlungsergebnissen im Suchtbereich, speziell Alkoholismus, außergewöhnlich umfangreich [18–20]. Pharmakologische Ansätze spielen in der Alkoholtherapie bislang eine untergeordnete Rolle [16, 24]. Obwohl der „Primary-care“-Bereich die Hauptlast in der Versorgung alkoholkranker Patienten trägt, haben sich in diesem Bereich nur wenige abstinenzorientierte Behandlungsansätze als wirksam erwiesen – in einer kürzlich vorgelegten, methodisch sehr sorgfältigen Network-Metaanalyse über 63 Studien (43 Interventionen) war dies überhaupt nur der Wirkstoff Acamprosat [1, 2]. Wirksame psychosoziale und psychotherapeutische Interventionen bei Alkoholabhängigkeit sind z. B. motivationale Therapien (z. B. „motivational interviewing“), kognitive und verhaltenstherapeutische Therapien (CBT), Kontingenzmanagement, Angehörigenarbeit und Paartherapie [14]. Sehr gut gesichert ist auch die Effektivität von Kurzzeitinterventionen („brief interventions“). In der aktuellen S3-Leitlinie werden u. a. Verhaltenstherapie, Kontingenzmanagement, motivationale Therapie und Rückfallprophylaxe als effektive Therapien genannt [5]. Akzeptierte Therapieziele sind sowohl Abstinenz als auch Verminderung der Trinkmenge (Harm-reduction-Strategie), wobei Letzteres bis heute nur als Ultima Ratio gilt [5]. Trotz dieser Vielzahl an Therapieansätzen und -studien ist festzustellen, dass in den letzten Jahren keine ganz neuen, innovativen Therapieansätze mehr etabliert werden konnten und ein großer Teil der Betroffenen weiter unbehandelt ist. Webbasierte Angebote können hier eine Lücke im Versorgungsangebot schließen, eine wertvolle Ergänzung etablierter Behandlungsansätze sein und neue Chancen in Diagnostik und Therapie von Alkoholgebrauchsstörungen eröffnen. Eine skandinavische Studie, die ein Onlineselbsthilfegruppenkonzept (kognitiv-behaviourales Konzept) nutzte, wurde von den Teilnehmern wegen der hohen Anonymität und Verfügbarkeit bevorzugt [8]. Eine andere skandinavische Studie nutzte eine asynchrone oder synchrone Chat-Möglichkeit mit einem Berater über einen zehnwöchigen Zeitraum, die einem reinen Selbsthilfesetting überlegen war [25]. Eine jüngst erschienene Metaanalyse wies auf die abstinenzförderliche Wirksamkeit internetbasierter Interventionen hin [26]. Insgesamt werden webbasierte Therapien sowohl „stand-alone“ als auch in Kombination mit konventionellen Ansätzen angeboten. Für CBT-basierte Interventionen liegt hier sogar eine Metaanalyse vor [15], wobei die Datenlage insgesamt noch überschaubar ist. Als relevante Faktoren nennt ein systematischer Review von Humphreys und Kollegen [10]: Vermittlung von Problemlösestrategien, Feedback und „self-monitoring“ des Verhaltens, Informationen über soziale und gesundheitliche Konsequenzen (also in gewisser Weise Psychoedukation) und „social comparison“. Eine direkte Vergleichbarkeit zu den hier dargestellten Daten lässt sich noch nicht herstellen.

Während sich die Vermittlung von Informationen und Aufklärung in webbasierten Medien insgesamt leicht darstellen lässt, sind interaktionelle Aspekte und Interventionen sehr viel schwieriger abbildbar, aber vermutlich entscheidend für den Erfolg der Intervention [8]. In den hier dargestellten Angeboten von OAmN ist dies teilweise implementiert, sowohl im öffentlichen als auch im Programmbereich. So baut OAmN interaktionelle Aspekte im öffentlichen Bereich z. B. dadurch ein, dass dazu aufgerufen wird, ein YouTube-Video zu posten, warum man an Weihnachten und Silvester nüchtern bleiben wird. Im Programmbereich wird beispielsweise dazu aufgefordert, die Onlinegruppe auf dem Weg in die Abstinenz mit einzubeziehen und Verbindlichkeit zu schaffen – etwa durch Nachrichten wie „Heute ist mein Tag 1“ oder dadurch, dass man in der Onlinegruppe Erfolgserlebnisse oder Bewältigungsstrategien für ein schwieriges Ereignis teilt.

Webbasierte Interventionen werden zunehmend eingesetzt, und ihre Evaluation hat sich zu einem aktiven Forschungsfeld entwickelt [9, 11–13, 23, 28]. Sie sind auf verschiedenen Ebenen und in verschiedenen Stadien der „Alkoholkarriere“ denkbar – zur Frühdiagnostik und -intervention, als eigentliche Therapie, evtl. auch komplementär dazu, zur Motivationsförderung oder in der Nachsorge. Vor allem aber können sich Betroffene austauschen und so auch ihre Selbstwirksamkeitserwartungen stützen. Vorteile solcher Interventionsebenen sind die niedrige Hemmschwelle, diese in Anspruch zu nehmen; leichte, z. T. ständige Verfügbarkeit und die Möglichkeit, selber aktiv zu werden. Auch Rückmeldungen und rasche Interventionen sind auf e‑basierten Medien möglich. Nachteilig ist das Fehlen oder zumindest der schwierige Aufbau empathisch getragener therapeutischer Beziehungen. Dennoch: Die sich abzeichnenden Perspektiven webbasierter Interventionen bei Alkoholgebrauchsstörungen sind erheblich. Dabei wird es nicht darum gehen, konventionelle therapeutische Inhalte und Materialien zu digitalisieren, sondern den neuen Medien entsprechende Interventionsformen und Materialien zu entwickeln. Bedarf und Interesse sowie Erreichbarkeit scheinen jedenfalls gegeben zu sein. Inwieweit gerade die Tatsache, dass OAmN nicht von der professionellen Suchthilfe entwickelt wurde, zu seiner Attraktivität beiträgt (niedrigere Hemmschwelle, leichtere Erreichbarkeit Betroffener, ebenso durch Peers), muss in zukünftigen Untersuchungen geklärt werden, ebenso die langfristigen Effekte.

Limitationen der Aussagekraft dieser Pilotstudie sind zum einen, wie bei jeder webbasierten Befragung, die begrenzte Kontrolle und Charakterisierung der Teilnehmer, was zu einem entsprechenden Teilnahmebias führen kann, zum anderen ist eine webbasierte Befragung auf die Ehrlichkeit (Bagatellisierungen des Alkoholkonsums, Tendenz zu sozial erwünschten Antworten) der Teilnehmer angewiesen; auch dies kann nicht kontrolliert werden. Jedoch ist zu vermuten, dass potenzielle Teilnehmer des Onlineangebots ein Problem in ihrem Trinkverhalten sehen, was das Bias der Bagatellisierung reduziert. Zudem bestand für die Teilnehmer eine erkennbare Anonymität, die den Probanden in der Datenschutzerklärung nochmals explizit zugesichert wurde.

Insgesamt ist darauf hinzuweisen, dass die Effekte der OAmN-Intervention zwar zeigen, dass bestimmte Personengruppen durch das Onlineangebot OAmN erreicht werden können, die durch das „klassische“ Suchthilfesystem nicht erreicht werden können und dass OAmN auch über eine gewisse Zeit zu Abstinenz führt; unklar ist jedoch, über welchen Zeitraum die Abstinenz anhält. Die Studie zeigt somit eine „Momentaufnahme“ und muss durch längere Verlaufsuntersuchungen ergänzt werden, um die Robustheit der Effekte aufzuklären.

## Fazit für die Praxis

- Webbasierte Interventionen scheinen ein interessantes und aussichtsreiches Medium für Menschen mit Alkoholproblem zu sein.
- Sie können eine Gruppe von Betroffenen erreichen, die trotz Alkoholproblem beruflich eingebunden und zumindest subjektiv „funktionsfähig“ ist.
- Diese Gruppe ist sich des problematischen Ausmaßes ihres Konsums bewusst und weist eine hohe Bereitschaft auf, das Problem anzugehen.
- Sie nutzt dazu aber größtenteils nicht die Angebote des etablierten Suchthilfesystems.
- Onlineangebote wie „Ohne Alkohol mit Nathalie“ (OAmN) können sowohl als Brücke als auch als komplementäres Angebot fungieren, um Hürden abzubauen, aufzuklären und frühzeitig zu helfen.

## Supplementary Information




