# Erratum zu: Nutzung und Akzeptanz webbasierter Angebote zur Alkoholabstinenz

**DOI:** 10.1007/s00115-023-01482-8

**Published:** 2023-04-26

**Authors:** Nathalie Stüben, Andreas G. Franke, Michael Soyka

**Affiliations:** 1 Klinik und Poliklinik für Psychiatrie und Psychotherapie, Ludwig-Maximilians-Universität München, Nußbaumstr. 7, 80336 München, Deutschland; 2 Hochschule der Bundesagentur für Arbeit, Mannheim, Deutschland; 3 Nathalie Stüben GmbH, Spinnereiinsel 3a, 83059 Kolbermoor, Deutschland

**Erratum zu:**

**Nervenarzt 2022**

10.1007/s00115-022-01385-0

Der offen und ausführlich dargelegte Interessenkonflikt der Erstautorin im in Ausgabe 1/2023 von *Der Nervenarzt* publizierten Beitrag „Nutzung und Akzeptanz webbasierter Angebote zur Alkoholabstinenz“ ist nun auch in der Interessenkonflikterklärung aufgeführt. Sie lautet nun:

## Interessenkonflikt.

Die Erstautorin N. Stüben ist Gründerin und Betreiberin des in der Studie untersuchten Web-Angebots. A.G. Franke und M. Soyka geben an, dass kein Interessenkonflikt besteht.

