# Supplementary material for: Acceptance and use of web-based interventions for alcohol abstinence
Source: Nervenarzt. 2022 Sep 13;94(1):1–7. [Article in German] doi: 10.1007/s00115-022-01385-0 (PMC9468517; doi:10.1007/s00115-022-01385-0)
Supplement: Supplementary file 1 [file 115_2022_1385_MOESM1_ESM.docx]

# Fragebogen Online-Umfrage zur Nutzung und Akzeptanz von OAmN

## Frage 1 richtete sich an alle Teilnehmer.

1. Warum nutzt Du die Angebote von OAmN? Bist Du selbst betroffen, also hast oder hattest Du ein Alkoholproblem?
   1. Ja
   2. Nein, aber eine mir nahestehende Person
   3. Nein, aber ich interessiere mich fürs Thema

## Die folgenden Fragen richten sich an alle, die mit **1a** geantwortet haben.

1. Aus welchen Gründen hast Du Alkohol konsumiert?
   *Mehrfachantworten möglich*
   1. Um Party zu machen
   2. Um meine Stimmung zu verbessern
   3. Um mich zu belohnen
   4. Um meinen Suchtdruck zu lindern
   5. Um mit meinem Stress umgehen zu können
   6. Um locker zu werden
   7. Um einschlafen zu können
   8. Um dazuzugehören
   9. Um meine Unsicherheit zu verstecken
   10. Um vergessen zu können
   11. Um körperliche Schmerzen zu lindern
   12. Um schwierige Gefühle zu betäuben
   13. Um abschalten zu können
   14. Aus Gewohnheit
2. Ist eine Alkoholabhängigkeit von einem Arzt oder einem Psychologen diagnostiziert worden?
   1. Ja
   2. Nein
3. Hast Du schon mal eine Therapie gemacht?
   1. Ja, eine
   2. Ja, mehrere
   3. Nein

### Nachfrage an 4a bzw. 4b

1. Welche Form von Therapie war das?
   1. Ambulant
   2. Stationär
   3. Beides

### Nachfrage an 4c

1. Planst Du eine Therapie?
   1. Ja
   2. Nein
2. Lebst Du schon abstinent?
   1. Ja
   2. Nein

## Die folgenden Fragen richten sich an alle, die mit **1a und 6a** geantwortet haben.

1. Wie lange lebst Du schon abstinent?
   1. Ganz frisch
   2. Über einen Monat
   3. Über drei Monate
   4. Über sechs Monate
   5. Über ein Jahr
2. Wie hast Du’s geschafft?
   *Offene Frage*
3. Wie oft hast Du vorher versucht, ganz mit dem Trinken aufzuhören?
   1. Ist mein erster Versuch
   2. Schon mehrfach
   3. Kann nicht mehr zählen
4. Hat Dich schon mal jemand auf Deinen Alkoholkonsum angesprochen?
   1. Nein
   2. Ja, Familie
   3. Ja, Freunde oder Bekannte
   4. Ja, Partner/in
   5. Ja, Arzt oder Therapeut
   6. Ja, im Beruf
5. Warst Du trotz Alkoholproblem berufstätig?
   1. Ja, in Vollzeit
   2. Ja, in Teilzeit
   3. Nein

### Nachfrage an 12a bzw. 12b

1. Wie gut hast Du Deiner Einschätzung nach trotz Deines Alkoholproblems beruflich funktioniert, so im Durchschnitt?
   1. Sehr gut
   2. Gut
   3. Befriedigend
   4. Ausreichend
   5. Mangelhaft
   6. Ungenügend
2. Warst Du schon vor der Coronavirus-Pandemie abstinent?
   1. Ja
   2. Nein

### Nachfrage an 14a

1. War es durch die Coronavirus-Pandemie schwerer für Dich, abstinent zu sein?
   1. Ja
   2. Nein

## Die folgenden Fragen richten sich an alle, die mit **1b oder 1c** geantwortet haben.

1. Trinkst Du Alkohol?
   1. Ja
   2. Nein

### Nachfrage an 16a

1. Wie oft und wie viel trinkst Du so im Schnitt, wenn Du Dir das letzte Jahr anschaust?
   *Offene Frage*
